# Estimating the prevalence of hematological malignancies and precursor conditions using data from Haematological Malignancy Research Network (HMRN)

**DOI:** 10.1007/s10552-016-0780-z

**Published:** 2016-06-28

**Authors:** Jinlei Li, Alex Smith, Simon Crouch, Steven Oliver, Eve Roman

**Affiliations:** Department of Health Sciences, University of York, Seebohm Rowntree Building, Heslington, York, YO10 5DD UK; Peking Union Medical College, Beijing, China

**Keywords:** Prevalence, Cancer registry, Epidemiology, Leukemia, Lymphoma, Myeloma

## Abstract

**Objective:**

Well-established cancer registries that routinely link to death registrations can estimate prevalence directly by counting patients alive at a particular point in time (observed prevalence). Such direct methods can only provide prevalence for the years over which the registry has been operational. Time-defined estimates, including 5- and 10-year prevalence, may however underestimate the total cancer burden, and compared with other cancers, there is a lack of accurate information on the total prevalence of hematological malignancy subtypes. Accordingly, we aimed to estimate prevalence (observed and total prevalence) of hematological malignancies and precursor conditions by clinically meaningful subtypes using data from the UK’s specialist population-based register, the Haematological Malignancy Research Network (www.hmrn.org).

**Methods:**

Observed and total prevalences were estimated from 15,810 new diagnoses of hematological malignancies from 2004 to 2011 and followed up to the 31 August 2011 (index data). Observed prevalence was calculated by the counting method, and a method based on modelling incidence and survival was used to estimate total prevalence. Estimates were made according to current disease classification for the HMRN region and for the UK.

**Results:**

The overall observed and total prevalence rates were 281.9 and 548.8 per 100,000, respectively; the total number of observed and total prevalent cases in the UK was estimated as 165,841 and 327,818 cases, as expected variation existed by disease subtype reflecting the heterogeneity in underlying disease incidence, survival and age distribution of hematological cancers.

**Conclusions:**

This study demonstrates the importance of estimating ‘total’ prevalence rather than ‘observed’ prevalence by current disease classification (ICD-O-3), particularly for subtypes that have a more indolent nature and for those that are curable. Importantly, these analyses demonstrate that relying on observed prevalence alone would result in a significant underestimation of the relative burden of some subtypes. While many of these cases may be considered cured and no longer being actively treated, people in this survivorship phase may have long-term medical needs and accordingly, it is important to provide accurate counts to allow for healthcare planning.

## Introduction

Cancer prevalence may be defined as the proportion of people in a population who have ever received a cancer diagnosis in the past and who are alive on a specified date—the index date. Cancer prevalence, which is generally estimated using data from cancer registries [1], provides information on the healthcare needs of cancer patients who are on long-term medication and/or who are being monitored at regular intervals. In addition, for cancers that can be cured, prevalence is used to estimate the size of the survivor population.

Well-established cancer registries that routinely link to death registrations can estimate prevalence directly by counting patients alive at a particular point in time. Such direct methods can, however, only provide prevalence for the years over which the registry has been operational: the term observed prevalence often being used to describe estimates derived from registries that have been established for shorter periods. In such cases, it is common practice to quote the length over which the registry has been functioning alongside the observed prevalence estimate. Likewise, 5- and 10-year prevalence estimates are commonly used to assess cancer burden: The former estimating patients diagnosed and ascertained in the previous 5 years and the latter in the previous 10 years. Time-defined prevalence estimates may, however, underestimate the total cancer burden, and in order to provide better guidance for healthcare planning, a variety of methods have been developed to estimate total prevalence (the proportion of the population alive on the index date who have ever received a diagnosis of the cancer). Total prevalence is usually estimated using models that incorporate incidence and survival [2–7].

Compared with other cancers, there is a lack of accurate information on the total prevalence of clinically meaningful hematological malignancy subtypes. This is partly because these complex cancers are diagnosed using a combination of histology, cytology, immunophenotype, cytogenetics, imaging and clinical data, and this range and depth of data are difficult for cancer registries to access systematically [8, 9]. Hence, the broad ICD-10 classification (leukemia, Hodgkin lymphoma, non-Hodgkin lymphoma and myeloma) has continued to be applied by many national registries [10], including the UK’s National Cancer Intelligence Network, the USA’s Surveillance, Epidemiology and End Results Program and the WHO’s International Agency for Research on Cancer [11–14].

Accordingly, the present study aims to estimate prevalence (observed prevalence and total prevalence) of hematological malignancies for clinically meaningful subtypes using data from the UK’s specialist population-based register, the Haematological Malignancy Research Network (www.hmrn.org) [15]. In addition, because hematological malignancies can be diagnosed at any age and survival patterns tend to differ between children and adults, adaptations to the statistical models used to estimate total prevalence were developed and applied.

## Methods

### Data

Data are from the UK’s population-based Haematological Malignancy Research Network (HMRN). Full details of its structure, data collection methods and ethical approvals have been described previously [16]. Briefly, within HMRN, patient care is provided by 14 hospitals, and as a matter of policy, all diagnoses are made in a single department that contains all relevant expertise and technologies to provide an integrated diagnostic service including histology, cytology, immunophenotyping and molecular cytogenetics. All diagnoses are coded to current WHO classification. Established in September 2004, HMRN collects information on all patients newly diagnosed with hematological malignancies in the study area (catchment population ~4 million, with ~2,200 new diagnoses per year) [15]. HMRN has Section 251 support under the NHS Act 2006, and all patients have full treatment, response and outcome data collected to clinical trial standards; all are ‘flagged’ for death registrations at the national Medical Research Information Service (MRIS).

### Calculating prevalence

The index date for prevalence calculations was taken to be 31 August 2011. Observed prevalence was calculated directly by counting the number of survivors newly diagnosed with a hematological cancer from 1 September 2004 to 31 August 2011, alive on the index date (31 August 2011). Total prevalence for each subtype was derived by applying an estimated correction factor, the completeness index (*R*), defined as the proportion of the total prevalence represented by the observed prevalence as described by Capocaccia and Angelis [17].

The calculation of the completeness index is based on incidence and survival models [17]. To accommodate the characteristics of hematological malignancies, a regression spline was used to estimate incidence for single ages. This nonparametric method makes a smoothing curve, which is not sensitive to the assumptions made for a parametric incidence function. For survival models, the Weibull function has previously been successfully applied to prevalence estimates [17–19]. Based on the Weibull function, the influence of age at diagnosis was described using spline and modelled with an exponential factor of survival function.

**Fig. 1:**
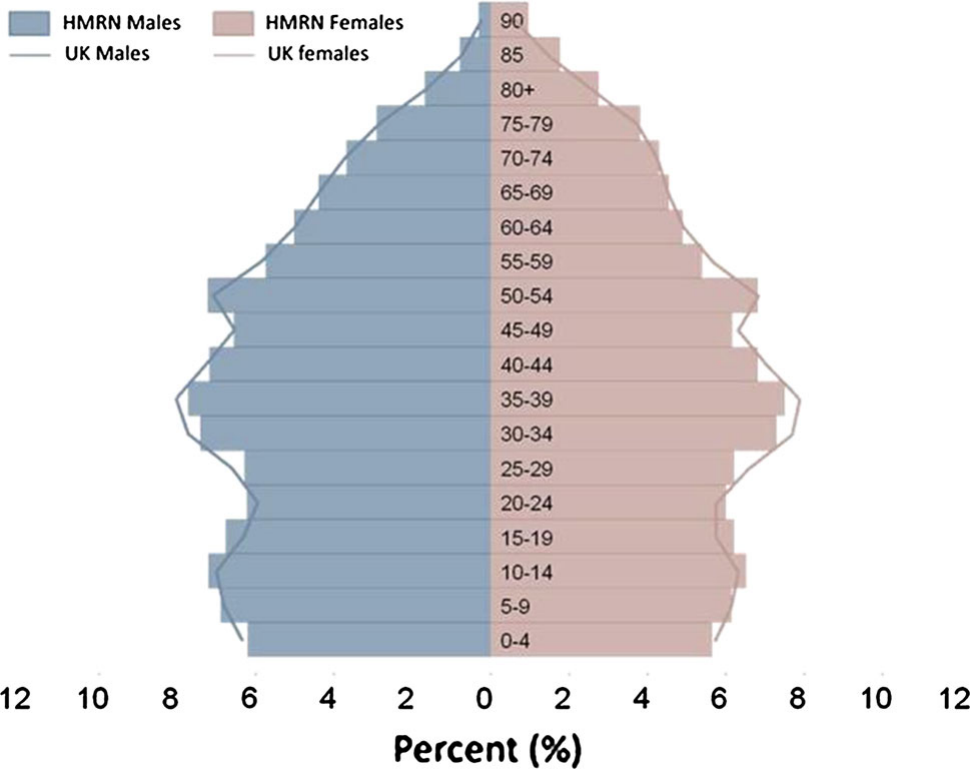
Population age and sex structure of the Haematological Malignancy Research Network (HMRN) region (*bars*) compared to the UK as a whole (*lines*), 2001 [16]

As the age and sex structure in the HMRN region mirrors that of the UK as a whole (Fig. 1), national prevalence was estimated by applying the HMRN rates to the UK population for both genders. All calculations were conducted using Stata 11.0 and R 3.0.1 software.

## Results

**Fig. 2:**
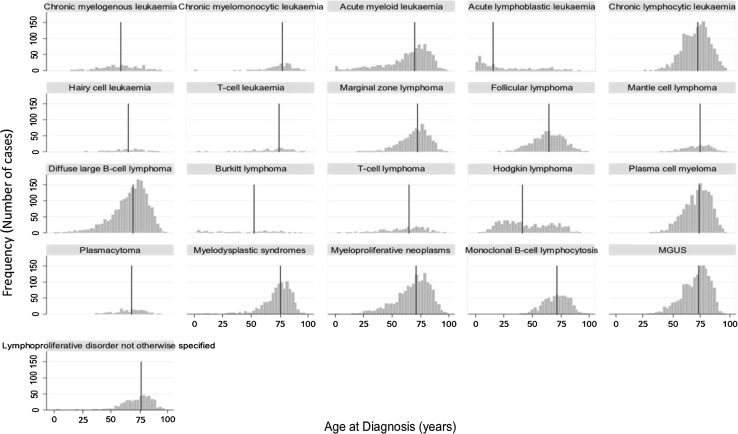
Age (years) at diagnosis (with *red lines* indicating median ages), distributions: Haematological Malignancy Research Network 2004–2011. *MGUS* monoclonal gammopathy of undetermined significance. (Color figure online)

There were 15,810 diagnoses of hematological malignancies from 2004 to 2011, of which 8,799 were among males (55.7 %) and 7,011 were females (44.3 %). The crude incidence rate for all hematological cancers combined was 63.2 per 100,000 per year and, as expected, incidence and survival varied by subtype: This variation is summarized in Table 1, where diagnoses are grouped according to their incidence magnitude (<2, 2–5, >5 per 100,000) and overall survival (<30, 30–70, >70 %). In addition to incidence and survival, age at diagnosis plays an important role in prevalence, and as with incidence and survival, hematological malignancies exhibit much greater variation than most other cancers. Indeed, with different subtypes dominating at different ages, hematological malignancy can be diagnosed at any age; the median age at diagnosis ranging from 15.3 years for acute lymphoblastic leukemia to 77.3 years for chronic myelomonocytic leukemia. Most subtypes had an older median diagnostic age (70.6 years for all hematological malignancies combined) (Fig. 2).

**Table 1:** Subtypes considered in this study, according to their incidence and survival categories^a^

Incidence (per 100,000)	Survival		
	Poor (5-year survival <30 %)	Medium (5-year survival 30–70 %)	Good (5-year survival >70 %)
Low (<2)	Chronic myelomonocytic leukemia	Acute lymphoblastic leukemia	Chronic myelogenous leukemia
	Mantle cell lymphoma	T cell leukemia	Hairy cell leukemia
		Burkitt lymphoma	
		T cell lymphoma	
		Plasmacytoma	
		Lymphoproliferative disorder not otherwise specified	
Medium (2–5)	Acute myeloid leukemia	Marginal zone lymphoma	Follicular lymphoma
	Myelodysplastic syndromes		Hodgkin lymphoma
			Monoclonal B cell lymphocytosis
High (>5)		Chronic lymphocytic leukemia	Myeloproliferative neoplasms
		Diffuse large B cell lymphoma	Monoclonal gammopathy of undetermined significance
		Plasma cell myeloma	

^a^Incidence and 5-year survival rates in HMRN from 2004 to 2011. Categories were made for this analysis only and cannot be generalized to other diseases or other data

Observed and total prevalence estimates (per 100,000) together with completeness indices (*R*) are presented in Table 2. The overall observed prevalence rate was 281.9 per 100,000, compared to a total prevalence rate of 548.8 per 100,000, the completeness index of 0.51 suggesting that around half of prevalent cases are not captured using observed prevalence. This varied by diagnostic subtype, ranging from 0.24 for Hodgkin lymphoma through to 0.90 for mantle cell lymphoma. As expected, subtypes with longer survival exhibited the largest differences between observed and total prevalence, whereas for those with poor survival, the estimates were much closer. For example, patients diagnosed with chronic myelomonocytic leukemia or mantle cell lymphoma during the study period had 5-year overall survival estimates <30 % (Table 1), and so completeness indices were high at 0.93 and 0.90, respectively. By contrast, patients diagnosed with chronic myelogenous leukemia or hairy cell leukemia, both of whom had 5-year overall survival estimates >70 % (Table 1), had comparatively low completeness estimates of 0.39 and 0.41, respectively.

**Table 2:** Observed and total prevalence (per 100,000) by sex: Haematological Malignancy Research Network 2004–2011

	Total			Male			Female		
	*R*	Observed	Total	*R*	Observed	Total	*R*	Observed	Total
Total	0.51	281.9	548.8	0.54	318	587.7	0.48	248.1	512.3
Leukemia	0.55	60.9	111.3	0.55	76.6	138.8	0.54	46.2	85.5
Chronic myelogenous leukemia	0.39	5.8	14.7	0.42	7.2	17.1	0.36	4.5	12.5
Chronic myelomonocytic leukemia	0.93	1.8	1.9	0.95	2.1	2.2	0.91	1.5	1.7
Acute myeloid leukemia	0.83	7.9	9.6	0.88	9.0	10.2	0.77	6.9	9.0
Acute lymphoblastic leukemia	0.39	5.6	14.5	0.41	6.8	16.5	0.35	4.5	12.6
Chronic lymphocytic leukemia	0.58	35.9	62.1	0.57	46.5	81.3	0.59	25.9	44.1
Hairy cell leukemia	0.41	2.0	4.9	0.39	3.4	8.6	0.54	0.8	1.5
T cell leukemia	0.53	1.9	3.6	0.58	1.7	3.0	0.51	2.1	4.2
Non-Hodgkin lymphoma	0.55	74.7	136.9	0.55	81.3	147.4	0.54	68.4	127.1
Marginal zone lymphoma	0.59	17.1	28.9	0.59	19	32.1	0.59	15.3	26.0
Follicular lymphoma	0.48	18.5	38.5	0.53	17.6	33.4	0.45	19.3	43.3
Mantle cell lymphoma	0.90	2.7	3.0	0.89	3.9	4.3	0.93	1.6	1.8
Diffuse large B cell lymphoma	0.57	31.5	55.1	0.56	34.5	61.2	0.58	28.7	49.4
Burkitt lymphoma	0.29	1.4	4.8	0.26	2.2	8.3	0.41	0.6	1.5
T cell lymphoma	0.54	3.6	6.6	0.53	4.3	8.1	0.56	2.9	5.2
Hodgkin lymphoma	0.24	17.3	72.4	0.27	19.8	73.3	0.21	15.0	71.5
Myeloma	0.79	23.8	30.1	0.78	29.4	37.5	0.80	18.6	23.1
Plasma cell myeloma	0.80	21.3	26.5	0.79	25.7	32.4	0.82	17.2	21.0
Plasmacytoma	0.71	2.5	3.5	0.72	3.7	5.1	0.69	1.4	2.1
Myelodysplastic syndromes	0.95	9.5	10.0	0.95	12.4	13.0	0.94	6.8	7.3
Other neoplasms of uncertain or unknown behavior	0.51	95.7	188.1	0.55	98.5	177.7	0.47	93.0	197.9
Myeloproliferative neoplasms	0.53	35.4	67.2	0.51	32.4	63.0	0.54	38.2	71.2
Monoclonal B cell lymphocytosis	0.50	16.5	32.9	0.58	18.2	31.3	0.43	14.9	34.5
Monoclonal gammopathy of undetermined significance	0.49	35.1	72.0	0.59	37.7	63.9	0.41	32.7	79.5
Lymphoproliferative disorder not otherwise specified	0.54	8.7	16.0	0.52	10.2	19.5	0.57	7.2	12.6

Index date of 31 August 2011

*R*: completeness index

In general, the difference between observed and total prevalence was greater for subtypes with more cases diagnosed at a young age. For example, although both Hodgkin lymphoma and follicular lymphoma have a medium incidence (2–5 per 100,000) and good survival (5-year survival >70 %) (Table 1), the completeness index of follicular lymphoma (0.48) was double that of Hodgkin lymphoma (0.24). This is because younger patients with Hodgkin lymphoma tend to be cured, resulting in a larger number of prevalent cases after middle age, while follicular lymphoma is rarely diagnosed before the age of 40 years.

**Fig. 3:**
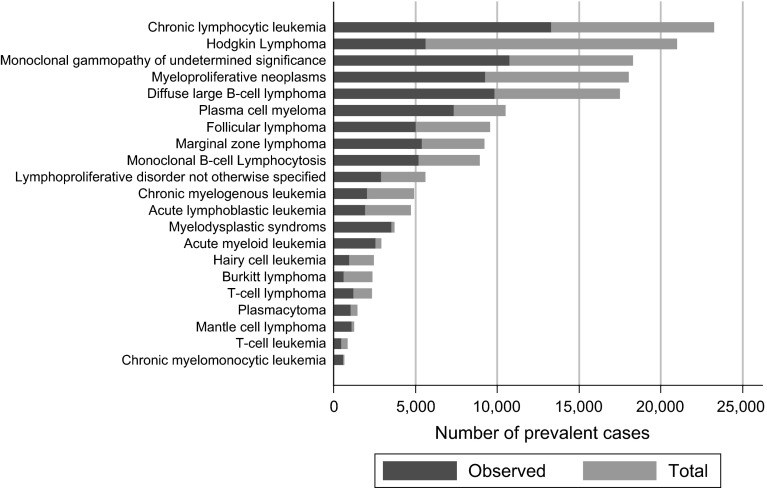
Observed and total prevalence cases for males in the UK on 31 August 2011. (Color figure online)

Observed and total prevalence estimates for the UK as a whole are presented in Figs. 3 (males) and 4 (females): observed prevalence (blue bars) and total prevalence (blue + red bar). Hematological malignancy subtypes are ranked in order of descending total prevalence. In total, the observed prevalence was estimated to be 165,841 cases and total prevalence 327,818 cases. Table 3 lists the UK observed and total prevalence estimates of the top five most prevalent hematological malignancies for males and females separately. Clearly, relying on observed prevalence alone would have resulted in a significant underestimation of the relative burden of some diseases. Observed prevalence, for example, ranks Hodgkin lymphoma as 6th for men and 8th for women, whereas total prevalence places it second for both genders. In other words, compared with observed prevalence, the relative contribution of Hodgkin lymphoma increases when longer prevalence periods are considered.

**Fig. 4:**
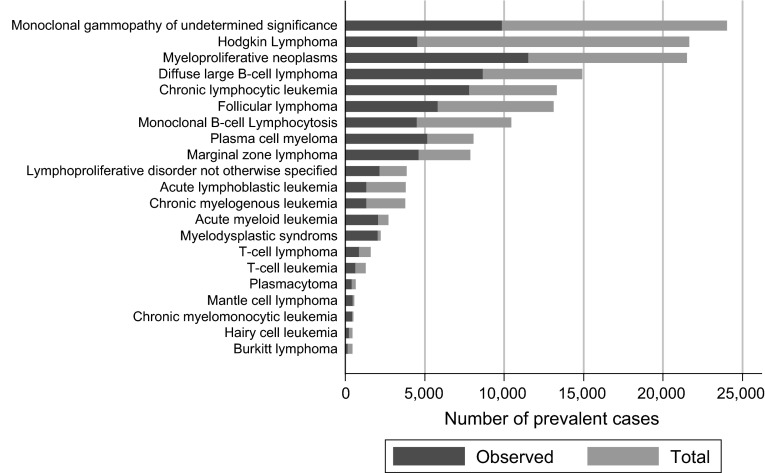
Observed and total prevalence cases for females in the UK on 31 August 2011. (Color figure online)

**Table 3:** Comparison of observed (7-year) and total prevalence of the top five hematological malignancies by gender in the UK

Observed		Total	
Disease	Prevalence	Disease	Prevalence
Male		Male	
Chronic lymphocytic leukemia	13,300	Chronic lymphocytic leukemia	23,222
Monoclonal gammopathy of undetermined significance	10,772	Hodgkin lymphoma	20,950
Diffuse large B cell lymphoma	9,847	Monoclonal gammopathy of undetermined significance	18,274
Myeloproliferative neoplasms	9,268	Myeloproliferative neoplasms	18,007
Plasma cell myeloma	7,352	Diffuse large B cell lymphoma	17,483
Female		Female	
Myeloproliferative neoplasms	11,536	Monoclonal gammopathy of undetermined significance	24,020
Monoclonal gammopathy of undetermined significance	9,878	Hodgkin lymphoma	21,608
Diffuse large B cell lymphoma	8,664	Myeloproliferative neoplasms	21,515
Chronic lymphocytic leukemia	7,827	Diffuse large B cell lymphoma	14,924
Follicular lymphoma	5,825	Chronic lymphocytic leukemia	13,316

## Discussion

This study is the first to estimate observed and total prevalence for hematological malignancies using up-to-date clinically meaningful disease classifications. The results suggest that at any one time, around 19,700 people in the study region are likely to be living with a prior diagnosis of a hematological malignancy or a recognized precursor condition (monoclonal gammopathy of uncertain significance or monoclonal B cell lymphocytosis): In total, this equates to around 327,800 people in the UK. After calculating total prevalence, the most prevalent malignancy in men was chronic lymphocytic leukemia and Hodgkin lymphoma in women.

Established in 2004, the HMRN’s population-based patient cohort provided an estimate of hematological malignancy prevalence that accounted for about half of the total (completeness index of 0.51). Consistent with expectations, the differences between total prevalence and observed prevalence estimates were typically seen in less fatal cancers that are commonly diagnosed at a younger age. For example, Hodgkin lymphoma generally has good survival, and total prevalence estimates exceed those of observed prevalence, while the difference between observed prevalence and total prevalence is slight for mantle cell lymphoma which has generally poor survival. Again, as expected, large differences between observed and total prevalence were also seen for precursor conditions. Information on 3-, 5- and 10-year prevalence is available on the study’s website (www.hmrn.org/statistics/prevalence) and has been published for the lymphomas and myeloid malignancies [20, 21].

Not only is the HMRN region similar to the UK as a whole in terms of its age and sex distribution, but it is also broadly similar by urban/rural and deprivation status [16]; accordingly, rates were not standardized by age and sex. Likewise, according to the 2011 [22] census, the proportion of HMRN’s population classified as white was the same as the UK as a whole (87 %). However, some ethnic groups are underrepresented in the region primarily the black ethnic group. For some hematological malignancies, such as myeloma, incidence and survival have been shown to vary by ethnicity with higher rates of both in black ethnic groups [14, 23, 24]. Accordingly, HMRN rates may underestimate myeloma prevalence in areas of the country with a higher proportion of black people [25].

This study assumes that the survival rate was constant over time; however, for some subtypes, there has been dramatic changes in outcomes due to the introduction of new treatments, for example, the introduction of tyrosine kinase inhibitors has transformed the survival in chronic myelogenous leukemia (CML) from a fatal disease in non-transplanted patients to one where patients can now achieve a near normal life span [26]. While CML is a rare disease (1 per 100,000), the utilization of current survival rates may lead to an overestimate in prevalence; accordingly, methods to estimate total prevalence need to be adapted to account for changes in outcome due to the introduction of novel therapies.

The major aim of this study was to estimate the prevalence of hematological malignancies and precursor conditions for clinically relevant diagnostic groups and explore the impact of calculating observed and total prevalence by current disease classification. For some subtypes, calculating observed prevalence would lead to an underestimation of the prevalent population, as cases diagnosed prior to the establishment of HMRN will not be captured. While many of these cases may be considered cured and no longer being actively treated, people in this survivorship phase may have long-term medical needs, and accordingly, it is important to provide accurate counts to allow for healthcare planning.
